# Mapping white matter structural and network alterations in betel quid-dependent chewers using high angular resolution diffusion imaging

**DOI:** 10.3389/fpsyt.2022.1036728

**Published:** 2022-12-05

**Authors:** Weiyuan Huang, Tao Liu, Huijuan Chen, Qingqing Fu, Lili Fu, Xiaolin Xu, Liting Liu, Yihao Guo, Priya S. Balasubramanian, Feng Chen

**Affiliations:** ^1^Department of Radiology, Hainan General Hospital, Hainan, China; ^2^Department of Geriatric Center, Hainan General Hospital, Hainan, China; ^3^Department of Radiology, Yueyang Central Hospital, Shanghai, China; ^4^Department of Electrical and Computer Engineering, Cornell University, Ithaca, NY, United States

**Keywords:** substance dependence, addiction, betel quid dependence, white matter, high angular resolution diffusion imaging, automated fiber quantification, anatomical network

## Abstract

**Background:**

To evaluate brain white matter diffusion characteristics and anatomical network alterations in betel quid dependence (BQD) chewers using high angular resolution diffusion imaging (HARDI).

**Methods:**

The current study recruited 53 BQD chewers and 37 healthy controls (HC) in two groups. We explored regional diffusion metrics alternations in the BQD group compared with the HC group using automated fiber quantification (AFQ). We further employed the white matter (WM) anatomical network of HARDI to explore connectivity alterations in BQD chewers using graph theory.

**Results:**

BQD chewers presented significantly lower FA values in the left and right cingulum cingulate, the left and right thalamic radiation, and the right uncinate. The BQD has a significantly higher RD value in the right uncinate fasciculus than the HC group. At the global WM anatomical network level, global network efficiency (*p* = 0.008) was poorer and Lp (*p* = 0.016) was greater in the BQD group. At the nodal WM anatomical network level, nodal efficiency (*p* < 0.05) was lower in the BQD group.

**Conclusion:**

Our findings provide novel morphometric evidence that brain structural changes in BQD are characterized by white matter diffusivity and anatomical network connectivity among regions of the brain, potentially leading to the enhanced reward system and impaired inhibitory control.

## Introduction

Betel quid is statistically consumed by more than 600 million people and is the fourth most prevalent self-administered psychoactive substance, after caffeine, alcohol, and nicotine (1). In mainland China, the incidence of betel quid dependence (BQD) among users ranged from 20 to 90% (2). Moreover, betel quid is considered to be a human carcinogen by the World Health Organization, and consequently, DSM-IV and ICD-10 dependence symptoms could appear with overdose (3, 4). BQD can potentially enhance the reward system and disrupt inhibitory control which can increase substance use habituation. The four primary natural alkaloids that makeup BQ are arecoline, Barcaldine, guvacoline, and guvacine (5). The major active component, arecoline, is known to assist in the release of dopamine by targeting M5 muscarinic acetylcholine receptors in the major reward network. Arecoline also binds to GABA receptors in the brain, and influences smooth muscles, leading to downstream psychoactive effects. Chronic BQ use is associated with reduced thinking and processing capacity, a higher level of satisfaction, amplified alertness and relaxation, and motor responses right after it (6). Repetitive BQ chewing dependency would make the positive sensations persistent. Common with other substance abuse patterns, individuals with BQD often experience tolerance, craving, betel quid-seeking behavior, and withdrawal (6). This is biologically linked to arecoline interaction with G protein-coupled receptors lowering receptor sensitivity (7).

It was observed that a link exists between betel quid abuse and alterations in brain function. Recently, Huang and co-workers have revealed that BQD is associated with functional changes in the brain regions in charge of reward, impulsivity, inhibitory control, and cognitive processing by using functional magnetic resonance imaging (fMRI) (8). Our previous study found that BQD showed increased connectivity from the anterior cingulate cortex (ACC) to the reward network (9). Other research studies have found that resting-state fMRI of BQD subjects compared to HC subjects have functional connectivity alterations in the ACC-brainstem, ACC-DMN connectivity, anterior default mode network (DMN), precuneus, insula, and hippocampus (10, 11). ACC was recognized as the most important region-mediated functional connectivity alteration during the process of betel quid addiction (9). ACC is believed to form part of an inhibitory system that exercises control over reward-related behavior (12), which is an important mechanism involved in the regulation of betel nut addiction. While numerous functional connectivity research has provided insights into the potential underlying mechanisms of BQD, the exact alterations of white matter (WM) diffusion characteristics and anatomical networks are still unknown and under-investigated.

The effects of chronic BQD on human brain WM structure and anatomical networks are not fully known. The available literature suggests that various forms of psychoactive substance dependence derive effects from underlying mechanisms in altering the structural connectivity of the brain (13–15), and we suppose a similar mechanism in the case of BQD. During the investigation of WM integrity alterations, as Yuan et al. (16) reported, a lower fractional anisotropy (FA) but higher mean diffusivity (MD) occurred in bilateral anterior thalamic radiation (ATR) of BQD individuals compared to HCs. However, a more holistic investigation of the diffusivity of other WM fibers and the anatomical network of WM was not evaluated.

While functional alterations as a result of BQD have been observed in previous studies, we hypothesize that BQD chewers also display morphometric and network alterations in a region-specific manner, with unknown consistency across subjects, regions, and characterization metrics. High angular resolution diffusion imaging (HARDI), one of diffusion MRI techniques, measures diffusion signals on a q-space sphere. It has been widely used in data collection process for structural connection analysis of human brain. Herein, the WM fiber and anatomical covariance network alterations based on HARDI data were thoroughly investigated using automated fiber quantification (AFQ) and graph theory in this work.

## Materials and methods

### Participants

Betel quid dependence (BQD) chewers were enrolled from primary candidates who chewed betel nut (from the areca palm *Areca catechu*) more than once a day and lasted more than 3 years in Hainan Province via the local advertisement or the department of psychology in our hospital during the study period. Throughout the same study period, community members were screened for age-matched healthy comparison individuals. This study was performed from January 2017 to January 2020.

Inclusion criteria for the BQD group were (1) age between 18 and 60, (2) diagnosis of BQD according to the BQD Scale (more than 4) (17) by a board-certified psychiatrist, and (3) Right-handed. Exclusion criteria for both BQD and healthy control (HC) groups were (1) any symptoms or signs of confusion, major medical disorders including kidney disease and chronic liver/lung disease, and/or malnutrition, (2) presence or history of neurological disorders, (3) presence or history of any mental disorders other than BQD or comorbid depressive disorders, including BQ induced persistent dementia, BQ induced amnestic disorder, or BQ withdrawal delirium, (4) presence or history of alcohol and/or tobacco addiction/dependence, (5) history of head injury, (6) any contraindications to magnetic resonance imaging (MRI) such as pacemakers, claustrophobia, or metal implants and (7) any lesion present in magnetic resonance (MR) images.

Each participant underwent a whole-brain MRI scan and a neuropsychological assessment with the Social Support Rate Scale (SSRS), Barratt Impulsiveness Scale, Hamilton Anxiety Scale-14 (HAMA-14), Hamilton Depression Scale-24 (HAMD-24), Montreal Cognitive Assessment (MoCA), Alcohol Use Disorders Identification Test, and Nicotine Dependence Test Scale for Smoker, all performed by neurologists blinded to the results of MR imaging results. SSRS includes 14 items with a total score of 40 and is one of the best effective instruments for assessing social support. Impulsivity is assessed with a 30-item self-report measure known as the Barratt Impulsiveness Scale (BIS-11). As a comprehensive measure including several cognitive tasks, the MoCA total score (range: 0–30) reflects global cognitive performance. To correct for educational effects found in the original study, participants with <12y of education receive a bonus point for corrections (18). We used a score of <26 to define cognitive impairment. Details of the neuropsychological assessment scales were showed in Supplementary material.

### MR data acquisition

The participants were scanned using a 3.0 T MRI scanner (Magneto Skyra, Siemens Erlangen, Germany), laying supine with the head snugly fixed by a belt and foam pads to minimize head motion, with a 32-channel standard head coil. 3D T1-weighted magnetization-prepared rapid gradient echo (MPRAGE) sagittal images: Repetition time/echo time (TR/TE) = 2530/2.98 ms, thickness/gap = 1.0/0.5 mm, flip angle = 7°, inversion time = 1100 ms, matrix = 256 × 256, field of view (FOV) = 256 mm × 256 mm. HARDI: Diffusion was measured along 64 non-collinear directions (b value = 1000 s/mm^2^), and an additional image without diffusion weighting (*b* = 0), TR/TE = 11600 ms/78 ms, matrix = 112 × 112, FOV = 224 mm × 224 mm, number of excitations = (*b* = 0, 10; *b* = 1000, 1), slice thickness = 2 mm with no gap.

### T1 image processing

**FIGURE 1:**
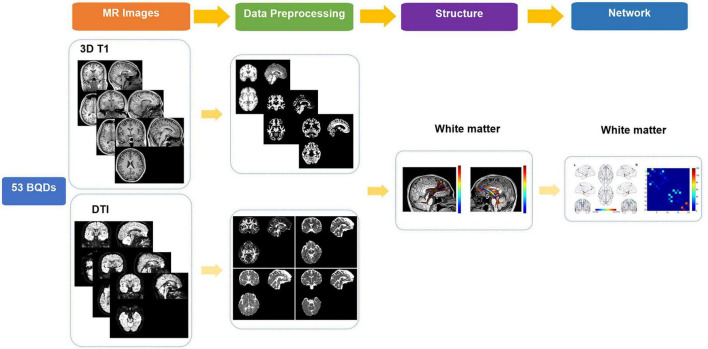
Flowchart of research on BQDs. BQDs, betel quid dependence chewers.

The workflow was shown in Figure 1. In this study, high-spatial-resolution T1-weighted MR imaging data were processed with voxel-based morphometry in statistical parametric mapping software (SPM12^1^) using Computational Anatomy Toolbox (CAT12^2^). At first, the anterior commissure was used to reorient all images and adjust origins after each image was reviewed for artifacts. Second, T1-weighted images were segmented into gray matter (GM), WM, and cerebrospinal areas; normalized to Montreal Neurologic Institute space; and resampled to a volume image resolution of 1.5 × 1.5 × 1.5 mm^3^.

### High angular resolution diffusion imaging processing

High angular resolution diffusion imaging (HARDI) data were coped with FMRIB’s diffusion toolbox (FMRIB’s Software Library, FSL5.0). Eddy current-induced distortion and motion artifacts in the HARDI dataset were corrected using affine alignment of each diffusion-weighted image to the b0 image. Each subject’s diffusion tensor matrix was calculated after skull-stripping, resulting in three pairs of eigenvalues and eigenvectors. Then, the intra-voxel metrics, including Fractional anisotropy (FA), mean diffusivity (MD), axial diffusivity (AD), and radial diffusivity (RD) maps were calculated based on the three eigenvalues.

### Automated fiber quantification

FSL performed segmentation and registration on 3D-T1 weighted images. AFQ software (19), a MATLAB toolbox, was utilized to detect and characterize 20 WM tracts in each subject’s brain using the pre-processed DTI and 3D-T1 weighted data. The original AFQ paper has specified the technical details and parameters (19); however, in this instance, the cingulum is subdivided into the cingulum cingulate and cingulum hippocampus. The four phases that make up the identification and quantification procedure are as follows: (1) tracing of whole brain fibers using deterministic tractography within WM masks for every individual; (2) A whole-brain fiber group is segmented into fascicles using an automated ROI approach. ROIs are defined in accordance with a previous study, and fiber tracts are refined in accordance with a probabilistic fiber tract atlas (JHU WM tractography atlas); (3) A fiber group is represented as a 3D Gaussian distribution before removal to identify its core, and stray fibers are removed that deviate from its core; (4) by weighing each fiber’s contribution to the measurement based on its distance from the track core, diffusion measurements are calculated along the path of the fiber group. Fiber tract profiles were defined by measuring diffusion along with the fiber tract core, which is a vector of Cues. For each individual, the tract profiles of FA, MD, AD, and RD at 100 equidistant nodes along each of the 20 tracts were ascertained to reflect their global WM status. The calculation formulas are as follows:


$$M\,D=\frac{\lambda_{1}+\lambda_{2}+\lambda_{3}}{3}$$

$$FA=\sqrt{\frac{2}{3}}\frac{\sqrt{{\left({\text{λ}}_{1}-\text{λ}\right)}^{2}+{\left({\text{λ}}_{2}-\text{λ}\right)}^{2}+{\left({\text{λ}}_{3}-\text{λ}\right)}^{2}}}{\sqrt{{\text{λ}}_{1}^{2}+{\text{λ}}_{2}^{2}+{\text{λ}}_{3}^{2}}}$$

$$A\,D=\lambda_{1}$$

$$R\,D=\frac{\lambda_{2}+\lambda_{3}}{2}$$

The 20 identified tracts included: bilateral thalamic radiation (TR), corticospinal tract (CST), cingulum cingulate (CCing), cingulum hippocampus (CHippo), inferior frontal-occipital fasciculus (IFOF), inferior longitudinal fasciculus (ILF), superior longitudinal fasciculus (SLF), uncinated fasciculus (UF), arcuate fasciculus (AF), Callosum Forceps Major (CFmajor) and Callosum Forceps Minor (CFminor).

### High angular resolution diffusion imaging anatomical network analysis

#### White matter anatomical networks construction

PANDA toolbox was applied to the diffusion metrics’ preprocessing and analysis. Atlas-based FACT deterministic fiber tracking was implemented with the pre-set motor-sensory network: (angle threshold = 45°FA threshold = 0.2).

The 20 nodes considering the DMN network were extracted from an automated anatomical labeling-90 (AAL-90) atlas (Table 1). The HARDI native-space cortex parcellations for each of the AAL atlas were constructed by using the inverse transformations. To construct the WM connectome matrix, deterministic fiber tracking was conducted to monitor WM connections for all possible pairs of nodes. Hence, A 20 × 20 white matter connectome matrix was developed for each subject, and each edge in the matrix was weighted by averaged Fiber Number (FN), Fractional Anisotropy (FA), and Matrix length (FL).

**TABLE 1:** 20 DMN regions from an automated anatomical labeling-90 (AAL-90) atlas.

1	3	Frontal_Sup_L	Superior frontal gyrus, dorsolateral	SFGdor.L
2	4	Frontal_Sup_R	Superior frontal gyrus, dorsolateral	SFGdor.R
3	23	Frontal_Sup_Medial_L	Superior frontal gyrus, medial	SFGmed.L
4	24	Frontal_Sup_Medial_R	Superior frontal gyrus, medial	SFGmed.R
5	29	Insula_L	Insula	INS.L
6	30	Insula_R	Insula	INS.R
7	31	Cingulum_Ant_L	Anterior cingulate and paracingulate gyri	ACG.L
8	32	Cingulum_Ant_R	Anterior cingulate and paracingulate gyri	ACG.R
9	37	Hippocampus_L	Hippocampus	HIP.L
10	38	Hippocampus_R	Hippocampus	HIP.R
11	59	Parietal_Sup_L	Superior parietal gyrus	SPG.L
12	60	Parietal_Sup_R	Superior parietal gyrus	SPG.R
13	61	Parietal_Inf_L	Inferior parietal, but supramarginal and angular gyri	IPL.L
14	62	Parietal_Inf_R	Inferior parietal, but supramarginal and angular gyri	IPL.R
15	67	Precuneus_L	Precuneus	PCUN.L
16	68	Precuneus_R	Precuneus	PCUN.R
17	85	Temporal_Mid_L	Middle temporal gyrus	MTG.L
18	86	Temporal_Mid_R	Middle temporal gyrus	MTG.R
19	89	Temporal_Inf_L	Inferior temporal gyrus	ITG.L
20	90	Temporal_Inf_R	Inferior temporal gyrus	ITG.R

#### Graph theoretical measures

With the assistance of the graph-theoretical network analysis toolkit (GRETNA^3^), the global and nodal topological metrics were determined by using the FN matrix. Only the fiber number reached 3 was included in the index calculation. The global characteristics metrics/properties [Assortativity, Hierarchy, Network Efficiency-Global (Eg), Network Efficiency-Local (Eloc), Small-World, Gamma, Lambda, Lp, Sigma, and Synchronization] and nodal characteristics metrics/properties (Betweenness Centrality, Degree Centrality, Nodal-Clustering Coefficient, Nodal Efficiency, Nodal Local Efficiency, Nodal Shortest Path) were calculated. These values were then used to measure how efficiently parallel information was exchanged over wide-scope and local brain regions. Small-world networks are rather efficient on both a global and local level, representing effective communication within both levels.

### Statistical analysis

For statistical analysis, pointwise comparisons of FA, MD, AD, and RD profiles were accomplished between healthy control individuals and BQD subgroups. Participant tract profiles were arranged in a single matrix. Using the FSL randomize program, all these matrices were fed into permutation-based statistical analysis with 5000 permutations, with age, gender, and education duration as covariates. The statistical results were subject to FDR correction for multiple comparisons and thresholded at *p* < 0.05.

To compare the anatomical networks based on HARDI, two-sample *t*-tests were conducted for global, nodal metrics and edge characteristics (*p* < 0.05, nodal metrics with FDR-corrected, edge characteristics with NBS-corrected) with and without covariates (GRETNA). The *Pearson* correlation analysis was used to measure the degree of association between BQD scales and FA, MD, AD, and RD variables with Bonferroni correlation.

## Results

### Sociodemographic and psychological characteristics

In this study, 79 BQD chewers were recruited. 26 BQD chewers were excluded due to comorbidity with alcohol addiction (9), smoking addiction (10), and 7 with both alcohol and smoking addiction. Finally, only 53 BQD chewers were included in the study. The sociodemographic and psychological characteristics of the BQD and HC groups are presented in Table 2. There was no significant difference in age, gender, and education duration between the BQD and HC groups. Moreover, only the Nicotine Dependence Test Scale for Smokers showed a statistical difference between the BQD and HC groups.

**TABLE 2:** Sociodemographic and psychological characteristics of BQD and HC groups.

	BQD	HC	P-value (two-tailed)
N	53	37	–
Gender (male/female)	38/15	24/13	0.214
Age (years)	38.15 ± 11.01	41.92 ± 11.63	0.122
Height (cm)	166.47 ± 7.62	164.41 ± 8.46	0.230
Weight (kg)	63.25 ± 10.43	61.30 ± 11.67	0.409
Years of education	12.83 ± 5.02	12.92 ± 2.68	0.922
Betel quid dependence scale	14.13 ± 8.25	–	–
Barratt impulsiveness scale	67.11 ± 8.72	67.49 ± 5.80	0.821
HAMA	1.58 ± 1.83	2.22 ± 1.87	0.115
HAMD	2.06 ± 2.22	2.59 ± 2.47	0.283
MOCA	26.32 ± 2.87	27.00 ± 3.15	0.292
SSRS	43.15 ± 8.20	40.84 ± 11.86	0.277
Alcohol use disorders identification test	1.83 ± 2.20	2.03 ± 2.33	0.684
Nicotine dependence test scale for smoker	1.57 ± 1.69	0.43 ± 1.07	0.001

HAMD, Hamilton Depression Scale; HAMA, Hamilton Anxiety Scale; MOCA, Montreal Cognitive Assessment; SSRS, Social Support Rate Scale.

### Results of diffusion metrics using automated fiber quantification analysis

**FIGURE 2:**
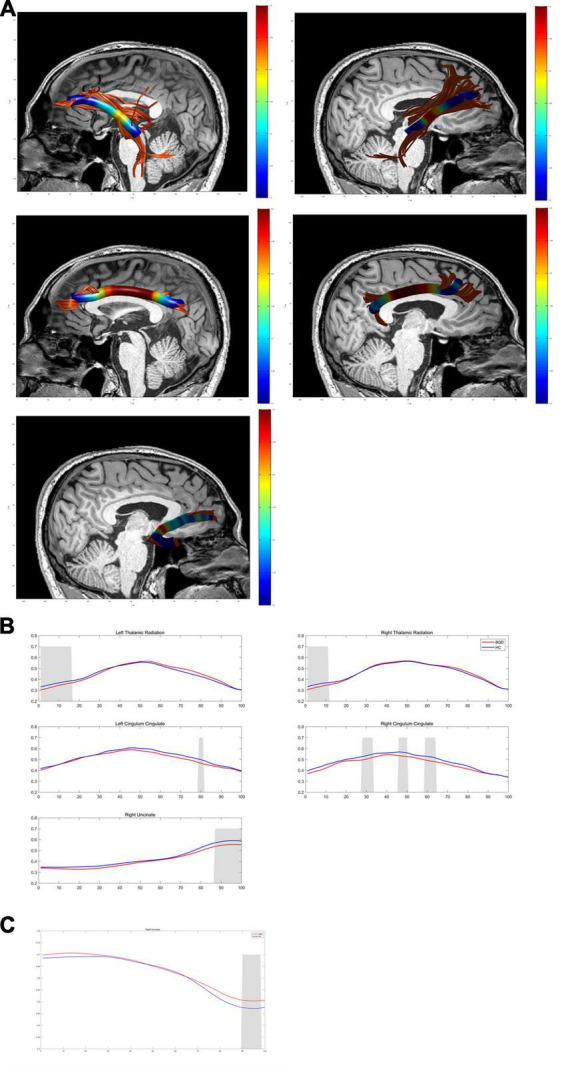
Diffusion metrics derived from DTI in the BQD group compared with the HC group using AFQ analysis. **(A)** Images show WM structural network based on DTI. **(B,C)** Curves of diffusion metrics in some brain regions of the BQD and HC group. DTI, diffusion tensor imaging; BQD, betel quid dependence; HC, healthy control; AFQ, automated fiber quantification; WM, white matter.

**FIGURE 3:**
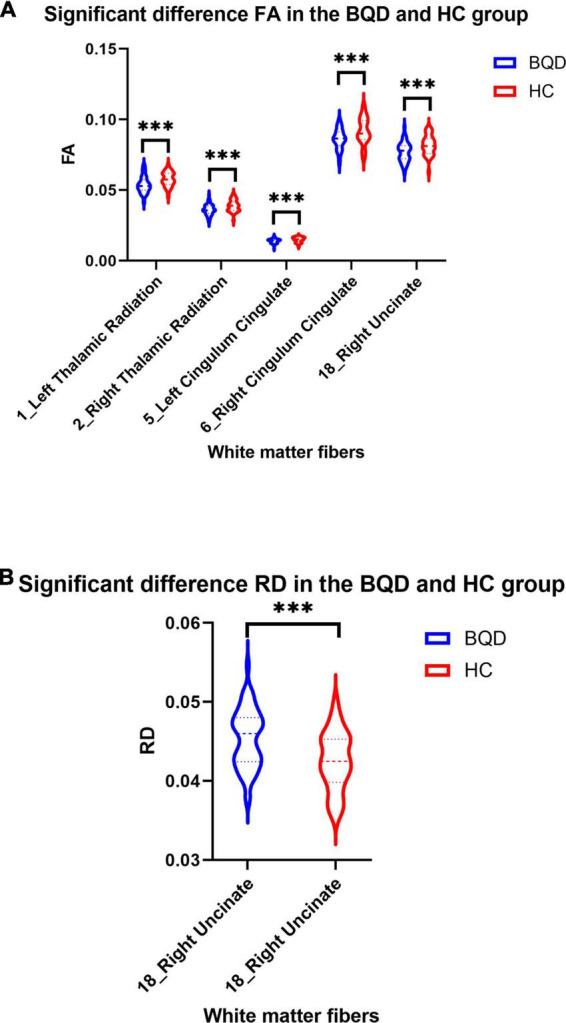
Violin plots show significant differences in FA **(A)** and RD **(B)** values of white matter fibers derived from DTI in the BQD and HC groups. Group difference: ^***^p < 0.001.

Except for bilateral cingulum hippocampus (CHippo) and arcuate fiber, all other sixteen WM tracts were traced successfully. Out of the sixteen WM tracts, the BQD have significantly lower FA values in the left and right cingulum cingulate, the left and right thalamic radiation, and the right uncinate with gender and education duration as covariates (Figures 2, 3). The BQD has a significantly higher RD value in the right uncinate fasciculus than the HC group without covariates (Figures 2, 3). MD and AD failed the FDR correction. After adding the Nicotine Dependence Test Scale as an additional covariate, the BQD have significantly lower FA values in the right thalamic radiation and higher RD value in the right uncinate fasciculus than the HC group. No significant correlation was found between HARDI quantitative parameters and BQD scales (*P* > 0.05).

### Results of white matter anatomical networks

#### Alterations in global network characteristics

**FIGURE 4:**
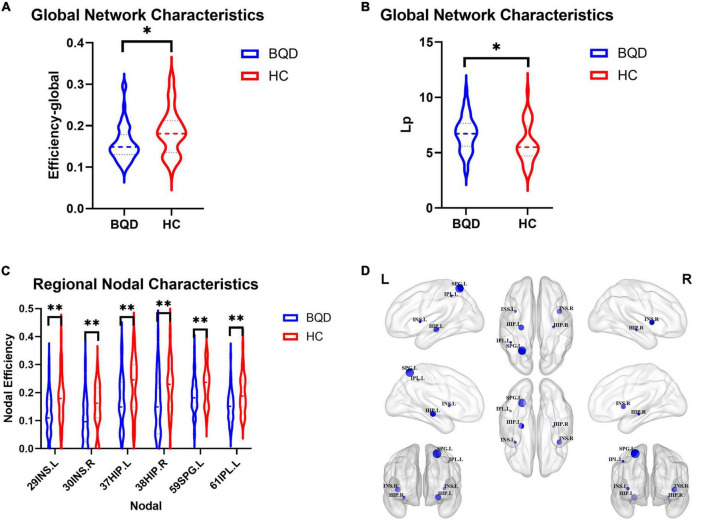
Violin plots show WM global network characteristics with statistically significant differences between the BQD and HC groups. BQD chewers had significantly less network efficiency-global **(A)**, and great Lp **(B)** at the global level. **(C)** The BQD group had lower nodal efficiency than the HC group in node 29 (INS.L), 30 (INS.R), 37 (HIP.L), 38 (HIP.R), 59 (SPG.L), and 61 (IPL.L). **(D)** The FN matrix of the right hippocampus (HIP.R) and precuneus (PCUN.R) was significantly different between the BQD and the HC groups in WM anatomical network. WM, white matter; BQD, betel quid dependence; HC, healthy control; FN, fiber number. Group differences: *p < 0.05, ^**^p < 0.01.

**FIGURE 5:**
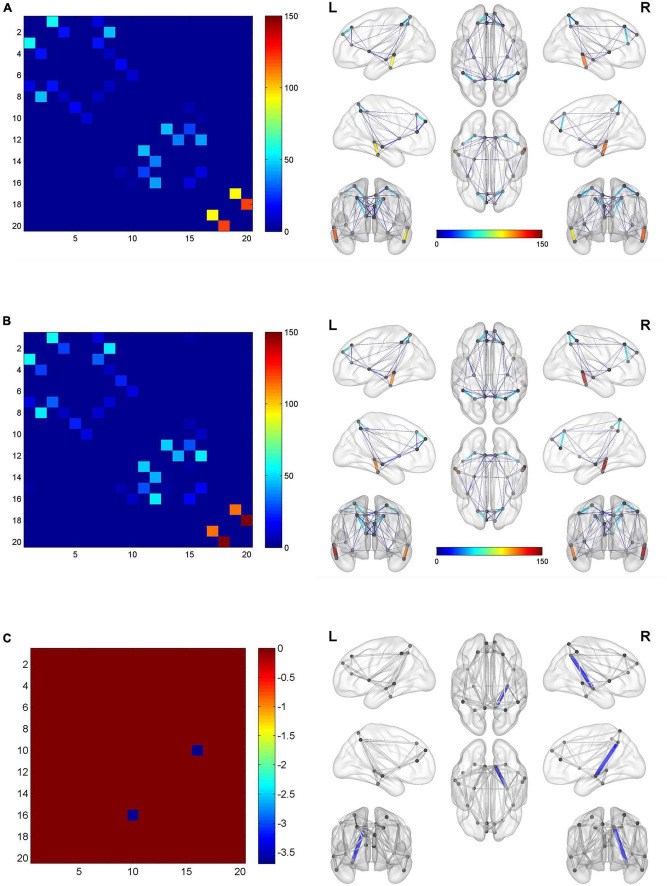
The global network connection of structural networks in the BQD group **(A)** and HC group **(B)**. Significant regions highlighted in **(C)**. BQD, betel quid dependence; HC, healthy control. The statistically significant differences were subject to FDR correction for multiple comparisons and thresholded at *p* < 0.05.

Figure 4 shows two global network metrics with statistically significant differences. BQD chewers had significantly lower Network Efficiency-global (*p* = 0.010) (Figure 4A), and higher Lp (*p* = 0.021) (Figure 4B) at the global level with gender and education duration as covariates (Figure 5). However, no significant differences (*p* > 0.05) were found in assortativity, hierarchy, Eloc, Gamma, Lambda, Sigma, Synchronization, and the small world at the global level with or without covariates.

Further, since the Nicotine Dependence Test Scale for Smokers showed a statistical difference between the BQD and HC groups, we have added the Nicotine Dependence Test Scale as an additional covariate in addition to age, gender, and education duration. Alterations in global network characteristics remain the same. BQD chewers had significantly lower Network Efficiency-global (*p* = 0.002) (Supplementary Figure 1A), and higher Lp (*p* = 0.005) at the global level (Supplementary Figure 1B). However, no significant differences (*p* > 0.05) were found in assortativity, hierarchy, Eloc, Gamma, Lambda, Sigma, Synchronization, and the small world at the global level with or without covariate.

#### Alterations in regional nodal characteristics

As shown in Figure 4, the BQD group had lower nodal efficiency than the HC group in Node 29 (INS.L), 30 (INS.R), 37 (HIP.L), 38(HIP.R), 59 (SPG.L), and 61 (IPL.L) with covariates (*p* ≤ 0.05, FDR-corrected) (Figures 4C,D).

After adding the Nicotine Dependence Test Scale as an additional covariate, as shown in Supplementary Figure 1, the BQD group had lower DegreeCentrality than the HC group in Node 29 (INS.L) and had lower NodalEfficiency than the HC group in Node 3 (SFGdor.L), Node 29 (INS.L), 30 (INS.R), 37 (HIP.L), 38 (HIP.R), 59 (SPG.L), 60 (SPG.R), 61 (IPL.L) and 67 (PCUN.L) with covariates (*p* ≤ 0.05, FDR-corrected).

#### Alterations in edge characteristics

**FIGURE 6:**
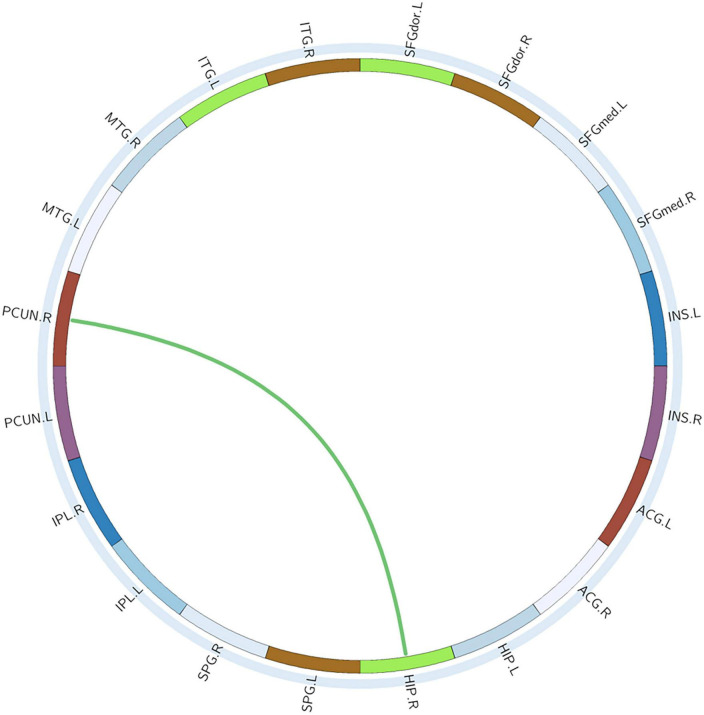
Circle graphs show WM anatomical brain network based on DTI with significant statistical results. The red line represents BQD > HC and the green line BQD < HC. DTI, diffusion tensor imaging; WM, white matter.

With or without the Nicotine Dependence Test Scale as an additional covariate, the FN matrix of the right hippocampus (HIP.R) and precuneus (PCUN.R) was significantly different between the BQD and the HC groups, and the number of fibers in BQD was significantly less than that in the HC group (Figures 5, 6).

## Discussion

This is the first study to perform a comprehensive investigation of brain WM structural and network changes in BQD chewers. We employed regional diffusion characteristics and WM anatomical networks based on HARDI, to investigate the morphometric change and network connectivity patterns of a population of BQD chewers. We observed a pattern of decreased anisotropy of WM fibers and WM anatomical network dysfunction in BQD chewers compared with HCs. These results confirm that BQD caused a morphometric change in WM structure and network dysconnectivity. The disturbance of the WM structural abnormality and disrupted network connectivity may be associated with the neurobiology of BQD chewers.

Betel quid dependence (BQD) chewers tend to have an enhanced reward system (11, 20, 21) and impaired inhibitory control (22, 23) according to the mechanism of BQ addiction. The reward areas in the brain include the basal ganglia, the limbic system, and parts of the prefrontal cortex (24). WM fiber diffusivity studies reveal that the BQD chewers have significantly lower FA values in the bilateral cingulum cingulate, bilateral thalamic radiation, and the right uncinate fasciculus than the HCs with covariates. In our work, the observation of lower FA and higher RD along the uncinate fasciculus of the right cerebral hemisphere is noteworthy. It was acknowledged that in BQD chewers, decreased FA with increased RD suggests an impairment in the size, myelination, or number of axons along with the fiber (25). The uncinate fasciculus is known as the largest WM bundle in charge of brain lateralization and interhemispheric communication. Demyelination or microstructure impairment along the uncinate fasciculus may affect neural communication across the hemispheres in complex ways, such as slower neural transmission speeds. The uncinate fasciculus regulates the pace of verbal information transmission and dynamically allocates processing resources, which are crucial for verbal memory (26). It projects into the frontal regions to temporal regions, including the amygdala. A rising number of studies have identified abnormal verbal information processing in substance abuse (26, 27). Chronic BQ use is associated with reduced thinking and processing capacity, a higher level of satisfaction, amplified alertness and relaxation, and motor responses right after it (26). We also found decreased FA values in many other regions (the bilateral cingulum cingulate, and bilateral thalamic radiation). Diffusion anisotropy decreases signify a loss of WM integrity, as evidenced by factors like the poorer extent of myelination, poorer fiber density, greater permeability, or more expanded extracellular space (28). Opiate addiction showed consistent results with ours’, showing decreased FA in the CCing and anterior thalamic radiation (29). Our previous and other BQD imaging studies confirmed that the BQD chewers have decreased spontaneous brain activity and decreased GM volume (30) in the right ACC, showing enhanced impaired inhibitory control. In the present study, we also showed decreased anisotropy in the right CCing, consistent with function alteration of ACC suggesting impaired inhibitory control in the BQD chewers. However, Weng et al. (31) reported enhanced diffusion anisotropy in the right ACC using generalized q-sampling imaging. Two possible explanations for the contradictory results are first, the different image protocol; and second, the ACC, which is thought to be critical for both inhibitory control and the amygdala-striatal reward system. Weng’s group indicated that chronic BQ chewing may not consistently worsen inhibitory control. It should be confirmed through more research if ACC exerts stronger inhibitory control on the amygdala-striatal reward system in BQDs. In future studies, patient or subject-specific usage side effects, symptoms, and behavioral changes during and following consistent BQ use will be investigated and correlated with specific structural and connectivity changes in the brain.

Thalamic radiation is the radiation of fibers connecting the thalamic nuclei and the cerebral cortex of the frontal lobe via the anterior limb of the internal capsule. Studies of different substances and behavioral addictions have examined addiction-related WM integrity using DTI and observed similar findings in thalamic radiation. Compared to healthy controls, BQD individuals showed statistically significantly lower FA and higher MD in bilateral anterior thalamic radiation (32). Our study complemented prior findings by providing further evidence for WM’s decreased transmission capacity in specific brain regions in BQD chewers. The results of this study thus lend credence to the theory that alterations in WM integrity, as well as those in the uncinate fasciculus, CCing, and thalamic radiation major, could contribute to an enhanced reward system and impaired inhibitory control characterized in BQD chewers.

Brain networks have demonstrated small-world properties in healthy populations, indicating a balance between local segregation and global integration among brain regions and networks at reduced wiring costs (33, 34). In this work, we reported a poorer network efficiency-global, greater Lp at the global level in the WM anatomical network of BQD chewers, which may represent a shift toward regularity in their anatomical brain networks and lead to the diminished organization of brain networks for efficient information transmission. Global efficiency reflects effective information transformation across remote regions, which is deemed to form the basis of many cognitive processes (35). Normalized Lp (lambda, λ) λ = Lpreal/Lprand, Lprand is the mean shortest path (36). In line with the results of previous studies on codeine addiction, chronic codeine abuse showed significantly higher Lp values, which indicates degradation of the small-world networks (36). The BQD group exhibited a decrease in global network efficiency coupled with an increased Lp in their brain network suggesting a decrease in transmission capacity/efficiency of information for the global network, the same as the result derived from a previous structural study in the BQD population (31). In line with the global level, we found a similar tendency in nodal level and edge level, with BQD chewers exhibiting a decreased local nodal and edge efficiency than HCs. Lower nodal efficiency was found in the bilateral insula, bilateral hippocampus, left superior parietal gyrus, and left inferior parietal gyrus. Most of these regions were the components of the mesocorticolimbic system. Lower nodal efficiency in mesolimbic might be associated with decreased functional connectivity from ACC to mesolimbic regions caused by BQD (9). Lower nodal efficiency was also observed in many brain regions in heavy smokers (37). We only involved BQD chewers lasting more than 3 years to investigate the change of structural network after a long-duration addiction. Our findings identified a decreased transmission efficiency for either the global or nodal network in chronic BQDs.

### Limitations

Several potential limitations of our study should be noted. Firstly, due to the cross-sectional nature of the study, potential differential changes in WM structure over the course of addiction in BQD remain to be directly established. Second, our sample was relatively small, which might reduce the power of the group comparison. Timepoint data across dependency to BQ is not yet established, and it is a challenge to entirely rule out other comorbid conditions or dependencies. Third, a combination of morphometric findings with abstinence treatment may enhance an understanding of the neurobiology of BQD and how these factors may affect neuroplasticity. Further, subject-specific dependency characteristics and sensitivities must be factored into the analysis.

## Conclusion

In conclusion, DTI revealed a comprehensive decreased anisotropy and regional increased RD of WM fibers in BQDs. Lower global efficiency, lower nodal efficiency, and higher Lp of the WM anatomical network were found in the BQDs. These findings confirmed that BQD morphometric alterations both occurred in WM fibers’ diffusivity and network connectivity, indicating that morphometric alterations of WM may be associated with the neurobiology of BQD chewers. Consistent with functional findings, our structural findings were characterized by an enhanced reward system and impaired inhibitory control in BQD chewers.

## Data availability statement

The original contributions presented in this study are included in the article/Supplementary material, further inquiries can be directed to the corresponding author.

## Ethics statement

The studies involving human participants were reviewed and approved by the RIB of Hainan General Hospital. The patients/participants provided their written informed consent to participate in this study.

## Author contributions

FC and WH: guarantors of the integrity of the entire study. WH, TL, HC, QF, LF, XX, LL, YG, PB, and FC: study concepts, study design, data acquisition, data analysis, interpretation, manuscript drafting, and manuscript revision. WH, TL, QF, LF, and XX: literature research. TL: clinical studies. QF, LF, XX, and LL: data acquisition. WH, HC, and YG: data analysis. HC and YG: statistical analysis. WH, TL, PB, and FC: manuscript editing. All authors approval of the final version of the submitted manuscript and agrees to ensure any questions related to the work are appropriately resolved.

## Footnotes

1
http://www.fil.ion.ucl.ac.uk/spm/

2
http://www.neuro.uni-jena.de/cat12/

3
http://www.nitrc.org/projects/gretna
