# Supplementary material for: Mapping white matter structural and network alterations in betel quid-dependent chewers using high angular resolution diffusion imaging
Source: Front Psychiatry. 2022 Dec 5;13:1036728. doi: 10.3389/fpsyt.2022.1036728 (PMC9760978; doi:10.3389/fpsyt.2022.1036728)
Supplement: Supplementary file 1 [file Data_Sheet_1.docx]

**Supplementary Materials**

**Details of the** **neuropsychological assessment scales**

SSRS includes 14 items with a total score of 40 and is one of the best effective instruments for assessing social support. The level of social support increases with a score. Low, medium and high levels of social support scored ratings ranging from 0-20, 20-30, and 30-40, respectively. Impulsivity is assessed with a 30-item self-report measure known as the Barratt Impulsiveness Scale (BIS-11). HAMA and HAMD to assess symptoms of anxiety and depression. The scores for no anxiety, suspected anxiety, definite anxiety, obvious anxiety, and severe anxiety were 0-7, 7-14, 14-21,21-29, and 29-64, respectively. The scores for low-level depression, medium-level depression, and high-level depression were 8-20, 20-35, and >35, respectively. As a comprehensive measure including several cognitive tasks, the MoCA total score (range: 0-30) reflects global cognitive performance. Participants with <12y of education receive a bonus point for corrections. We used a score of <26 to define cognitive impairment.

**Supplementary Figure1**


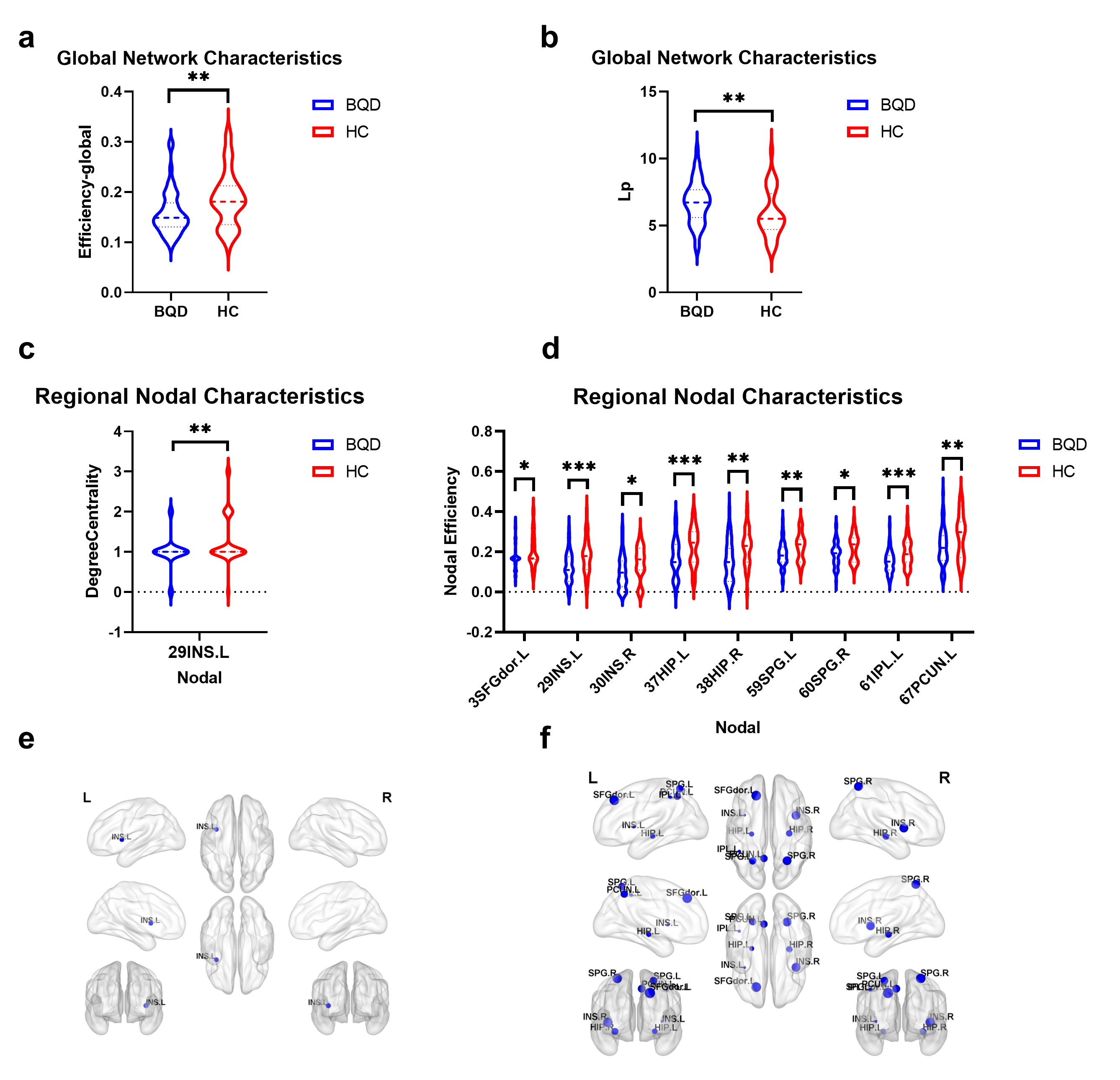
Caption. Violin Plots show WM global network characteristics with statistically significant differences between the BQD and HC groups, after adding the Nicotine Dependence Test Scale as an additional covariate. BQD chewers had significantly less Network Efficiency-global (a), and great Lp (b) at the global level. (c) The BQD group had lower DegreeCentrality than the HC group in Node 29 (INS.L). (d) The BQD group had lower Nodal Efficiency than the HC group in Node 3 (SFGdor.L), Node 29 (INS.L), 30 (INS.R), 37 (HIP.L), 38(HIP.R), 59 (SPG.L), 60 (SPG.R), 61 (IPL.L) and 67 (PCUN.L) with covariates (*p*≤0.05, FDR-corrected). (e) and (f) showed the location of significant difference nodes of DegreeCentrality and Nodal Efficiency in the WM anatomical network.

WM, white matter; BQD, betel quid dependence; HC, healthy control.

Group differences: **p*＜0.05; ***p*＜0.01, ****p*＜0.001.
